# The Impact of ioMRI on Glioblastoma Resection and Clinical Outcomes in a State-of-the-Art Neuro-Oncological Setup

**DOI:** 10.3390/cancers15143563

**Published:** 2023-07-10

**Authors:** Wei Zhang, Sebastian Ille, Maximilian Schwendner, Benedikt Wiestler, Bernhard Meyer, Sandro M. Krieg

**Affiliations:** 1 Department of Neurosurgery, School of Medicine, Klinikum rechts der Isar, Technical University of Munich, 81675 Munich, Germany; wei.zhang@tum.de (W.Z.); maximilian.schwendner@tum.de (M.S.); bernhard.meyer@tum.de (B.M.); 2 Department of Neuroradiology, School of Medicine, Klinikum rechts der Isar, Technical University of Munich, 81675 Munich, Germany; b.wiestler@tum.de

**Keywords:** extent of resection, glioblastoma, gross total resection, intraoperative MRI, Karnofsky performance scale, progression-free survival

## Abstract

**Simple Summary:**

The relationship between the implementation of intraoperative MRI and progression-free survival of glioblastoma has been questioned in a few meta-analyses, lacking analysis of large studies based on real-world data in contemporary neurosurgery. The present study supports the evidence that the use of intraoperative MRI in modern neurosurgery severely decreases residual tumor volume and considerably helps to achieve comparable progression-free survival, even in patients with unexpected residual tumor after initial resection before intraoperative MRI.

**Abstract:**

Intraoperative magnetic resonance imaging (ioMRI) aims to improve gross total resection (GTR) in glioblastoma (GBM) patients. Despite some older randomized data on safety and feasibility, ioMRI’s actual impact in a modern neurosurgical setting utilizing a larger armamentarium of techniques has not been sufficiently investigated to date. We therefore aimed to analyze its effects on residual tumor, patient outcome, and progression-free survival (PFS) in GBM patients in a modern high-volume center. Patients undergoing ioMRI for resection of supratentorial GBM were enrolled between March 2018 and June 2020. ioMRI was performed in all cases at the end of resection when surgeons expected complete macroscopic tumor removal. Extent of resection (EOR) was performed by volumetric analysis, with GTR defined as an EOR ≥ 95%, respectively. Progression-free survival (PFS) was analyzed through univariate and multivariate Cox proportional regression analyses. In total, we enrolled 172 patients. Mean EOR increased from 93.9% to 98.3% (*p* < 0.0001) due to ioMRI, equaling an increase in GTR rates from 78.5% to 93.0% (*p* = 0.0002). Residual tumor volume decreased from 1.3 ± 4.2 cm^3^ to 0.6 ± 2.5 cm^3^ (*p* = 0.0037). Logistic regression revealed recurrent GBM as a risk factor leading to subtotal resection (STR) (odds ratio (OR) = 3.047, 95% confidence interval (CI) 1.165–7.974, *p* = 0.023). Additional resection after ioMRI led to equally long PFS compared to patients with complete tumor removal before ioMRI (hazard ratio (HR) = 0.898, 95%-CI 0.543–1.483, *p* = 0.67). ioMRI considerably reduces residual tumor volume and helps to achieve comparable PFS, even in patients with unexpected residual tumor after initial resection before ioMRI.

## 1. Introduction

Intraoperative magnetic resonance imaging (ioMRI) has frequently been utilized for glioblastoma (GBM) resection over the past decade [1,2,3,4,5]. ioMRI can reportedly update image-guided neuronavigation intraoperatively, and thus help to potentially increase the extent of resection (EOR) [4,5,6]. However, these studies are based on data acquired from 2006 on, and often focus on an intraoperative setup providing ioMRI, while not applying advanced image fusion, neuronavigation, or preoperative and perioperative mapping and neuromonitoring [4,5,6]. A higher EOR is considered to be an independent factor associated with prolonged progression-free survival (PFS) [7]. Studies have shown that gross total resection (GTR) can significantly prolong PFS in patients suffering from GBM [8,9,10,11,12,13]. Numerous factors correlate with clinical outcomes of GBM, including tumor characteristics, patient age, patient performance status, and adjuvant therapy (Tx) [14,15,16,17]. Furthermore, ioMRI’s value and efficiency in prolonging the PFS of high-grade gliomas has frequently been discussed in recent studies after initial trials were performed in the absence of a modern armamentarium [3,18,19].

Therefore, ioMRI’s role in the resection of malignant brain tumors in a modern neurosurgical setting, including preoperative brain mapping, intraoperative neuromonitoring and mapping, advanced neuronavigation updated by intraoperative imaging, and fluorescence-guided surgery requires discussion and reconsideration [20,21].

In the present study, we aimed to analyze the impact of ioMRI on EOR, identify subgroups that benefit most from ioMRI, and correlated additional resection after ioMRI with clinical and neurological patient outcome, based on data of a single high-volume center using the whole modern armamentarium of surgical neurooncology.

## 2. Materials and Methods

### 2.1. Ethics Approval

The local ethics board approved the study (registration number: 336/17, 192/18). We conducted the study in accordance with the Declaration of Helsinki and the PROCESS statement. All patients consented to the procedure.

### 2.2. Data Collection

Patients undergoing supratentorial tumor resection between March 2018 and June 2020 at our institution were prospectively enrolled in a database. Data analysis was performed retrospectively with a cut-off time for follow-up in December 2021. Patients included in this study were histologically diagnosed with GBM and underwent ioMRI for intraoperative control of EOR. Postoperative MRI within 48 h and at least one valid follow-up MRI imaging was performed in all cases. Patients undergoing staged operations and patients younger than 18 years were excluded.

Patient characteristics, surgical data, duration of ioMRI, as well as imaging data were acquired. Regarding clinical outcomes, the Karnofsky performance status scale grade (KPS) on the fifth postoperative day (POD5) and in the third postoperative month (POM3) was assessed. Time from surgical resection to GBM progression was recorded. Each patient’s preoperative and postoperative motor status, as well as language function, graded according to the Aachener Aphasia Test, was evaluated and documented.

### 2.3. Standard Treatment and Tumor Progression Determination

Patients diagnosed with GBM were treated according to the European Association of Neuro-Oncology (EANO) guidelines [22,23]. Adjuvant therapy was individually adapted after multidisciplinary tumorboard discussion. Neurological examination and follow-up MRI were provided routinely every three months and in case of new symptoms. Multidisciplinary specialists determined the definition of tumor progress (TP) based on MRI findings and clinical features according to the Response Assessment in Neuro-Oncology (RANO) criteria [24].

### 2.4. MRI Scan and Tumor Resection

All patients underwent a structural MRI scan (3T MRI scanner, Achieva, Philips Medical Systems) prior to surgery according to the standard MRI protocol, including a 3D gradient echo sequence with intravenous contrast agent. Preoperative navigated transcranial magnetic stimulation (nTMS)-based motor and language mappings, as well as diffusion tensor imaging (DTI) tractography, were performed in 144 and 55 cases, respectively [25]. Intraoperative neuromonitoring and awake language mapping were performed depending on tumor location. ioMRI was performed using a high-field MRI scanner (3T MR scanner Ingenia, Philips Medical System, Netherlands B.V., Eindhoven, Netherlands) and updating the neuronavigation system after the end of the first resection. T1-weighted imaging with and without contrast agent was routinely performed in all cases. After the operating surgeon reviewed the ioMRI scan, additional resection was performed in case of tumor residuals considered resectable. In all cases, a postoperative MRI scan was performed within 48 h after surgery.

### 2.5. Tumor Volumetric Analysis

To measure tumor volume on preoperative MRI and residual volume on ioMRI and postoperative MRI, contrast-enhancing tumor volume was delineated using a semiautomatic contouring tool (SmartBrush, version 3.0.0.92, Brainlab AG, Munich, Germany). In few selected cases of lesions showing only minor contrast enhancement, additional sequences on MRI and PET (positron emission tomography) imaging were reviewed for tumor volume delineation. In addition, the EOR was calculated, and cases were divided into GTR and subtotal resection (STR), with GTR being defined as EOR ≥ 95% [5,26].

### 2.6. Statistical Analysis

Data were analyzed using GraphPad Prism (version 9.0, GraphPad Software Inc., San Diego, CA, USA) and SPSS (version 21.0, International Business Machines Corp., Armonk, NY, USA). *T*-test was used to compare continuous variables, including tumor volume and extent of resection. Fisher’s exact test was used to compare frequency variables, including GTR/STR and with/without additional resection after ioMRI. PFS was analyzed using univariate and multivariate Cox proportional regression analyses. Variables of interest, including the impact of with/without additional resection on PFS and the impact of POD5/POM3 KPS on PFS were also analyzed using Kaplan–Meier survival analysis. Logistic regression was used to probe factors leading to STR. Correlation analysis was conducted to study the impact variables on KPS on the fifth postoperative day (POD5-KPS) and in the third postoperative month (POM3-KPS), respectively. The level of significance was set at *p* < 0.05.

## 3. Results

### 3.1. General Information

A total of 277 resections assisted by ioMRI were consecutively performed in 251 patients in our department from March 2018 to June 2020 and analyzed in this study. A total of 180 out of 277 cases were histopathologically diagnosed with GBM. We considered 172 of those 180 cases eligible for further analysis, as eight cases were lost to follow-up after resection, with no follow-up MRI data after discharge available. The mean follow-up was 8.1 months, with a maximum length of follow-up of 30.6 months. The mean duration of the ioMRI-related pause in surgery was 45.2 ± 8.5 min. Further general information is presented in Table 1.

Preoperative KPS and KPS on POD5 were available in all 172 cases, while KPS in POM3 was available in 117 cases. A total of 24 out of 117 cases (20.5%) showed TP within POM3. Postoperative adjuvant radiotherapy (RTx) and/or chemotherapy (CTx) was performed before TP in 103 cases. Forty-six cases received no RTx or CTx before TP, while 23 cases had no record of Tx before lost to follow-up (FU). The distributions of cases among adjuvant treatments were comparable between the groups with and without resection after ioMRI (*p* = 0.51) (Table 2).

### 3.2. Volumetric Analysis

**Figure 1:**
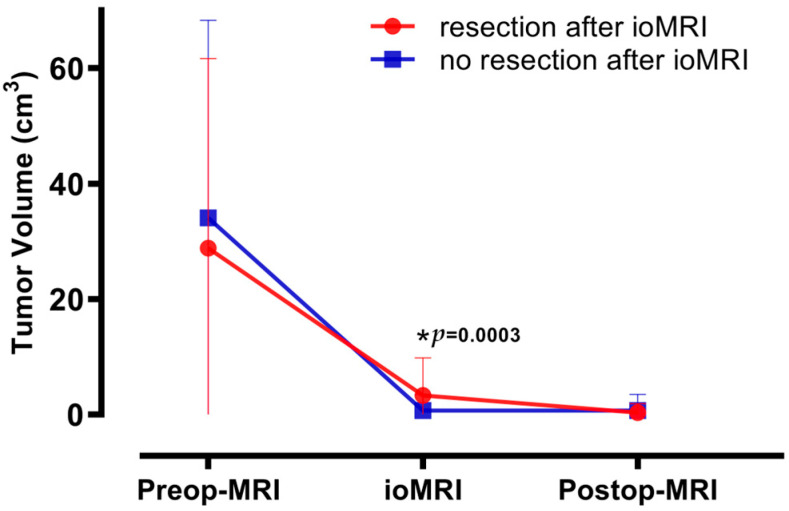
This figure displays the mean glioblastoma volume and standard deviation measured at preoperative MRI (Preop-MRI), intraoperative MRI (ioMRI), and postoperative MRI (Postop-MRI). We separately illustrate and compare cases with and without additional resection after ioMRI. *T*-testing showed *p*-values of 0.37 for Preop-MRI, 0.0003 (marked with *) for ioMRI and 0.41 for Postop-MRI.

Regarding volumetric analysis, an EOR of 93.9 ± 14.3% on ioMRI and 98.3 ± 8.2% on postoperative MRI was accomplished (Table 3, Figure 1).

Forty-three cases underwent additional resection after ioMRI. Sixteen cases showed GTR with a tiny residual tumor, and 27 cases showed STR. In three cases, additional resection was limited, as intraoperative monitoring suggested residual tumor adjacent to functional areas, with two cases failing to achieve GTR. The GTR rate drastically increased, from 37.2% to 95.3% (*p* < 0.0001), with residual tumor volume decreasing from 3.3 ± 6.5 cm^3^ to 0.3 ± 1.6 cm^3^ at postoperative MRI (*p* = 0.0028) among those 43 cases with additional resection after ioMRI.

In 129 cases, no additional resection was performed after ioMRI. In 117 cases, complete tumor resection was achieved. In 12 cases of residual tumor on ioMRI, including two instances of GTR, further resection was abandoned due to a high risk of functional deficits. The mean residual tumor volume was 0.7 ± 2.8 cm^3^ in patients without additional resection.

Overall, the GTR rate increased from 78.5% to 93.0% (*p* = 0.0002). EOR increased from 93.9% before ioMRI to 98.3% (*p* < 0.0001), with residual tumor volume decreasing from 1.3 ± 4.2 cm^3^ to 0.6 ± 2.5 cm^3^ (*p* = 0.0037).

### 3.3. Impact Factors Leading to Subtotal Resection

The aim of surgery was to achieve complete resection. However, according to the surgeon’s evaluation, tumor resection was terminated in 15 of 172 cases before achieving intraoperative complete macroscopic tumor resection. In 11 cases, tumor residues were located close to eloquent brain structures (insular lobe and basal ganglia in five cases, corticospinal tract as indicated by tractography in five cases, large vessels in one case). In four cases, resection was terminated due to unstable and decreasing motor-evoked potentials in neuromonitoring. To detect factors leading to STR, we therefore included 157 cases with complete tumor resection and defined those as completely resectable. We observed an EOR of less than 95% on ioMRI in 24 cases. In univariate analysis, recurrent GBM (*p* = 0.0013) and tumor volume < 15 cm^3^ (*p* = 0.0032) were risk factors contributing to STR (Table 4). After logistic regression (X^2^ = 15.137, *p* = 0.001 for Omnibus tests), we confirmed recurrent GBM (odds ratio (OR) = 3.047, 95% confidence interval (CI) 1.165–7.974, *p* = 0.023) and tumor volume less than 15 cm^3^ (OR = 3.031, 95% CI 1.062–8.651, *p* = 0.038) as independent risk factors leading to STR (Table 4).

### 3.4. Progression-Free Survival

**Figure 2:**
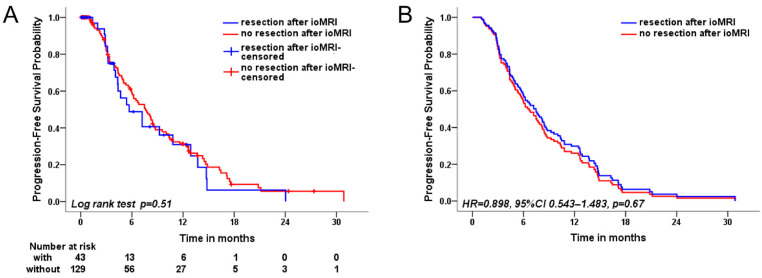
Kaplan–Meier curves and adjusted Kaplan–Meier curves of cases with and without additional resection. This figure illustrates the Kaplan–Meier survival curves in univariate analysis (**A**) and the adjusted Kaplan–Meier survival curves after multivariate analysis (**B**). We compared the progression-free survival of patients with additional resection after intraoperative MRI (blue) to patients without additional resection (red).

The overall median PFS of patients suffering from GBM in this large series was 7.5 months. We included age, gender, tumor characteristics regarding primary tumor manifestation or recurrence, tumor volume, necessity of resection after ioMRI, EOR, and Tx before TP in the Cox proportional regression analyses (Table 5). Testing of the proportional hazards assumptions was carried out for all involved variables. The number of events was 108, and events per variable were 13.5. In the univariate analysis, higher age (HR = 1.017, 95%CI 1.001–1.033, *p* = 0.037), worse preoperative KPS (HR = 0.981, 95%CI 0.966–0.995, *p* = 0.0081), and no Tx before TP (HR = 0.475, 95%CI 0.295–0.766, *p* = 0.0023) were identified as leading risk factors of TP. In the multivariate analysis, worse preoperative KPS (HR = 0.983, 95%CI 0.967–0.998, *p* = 0.031), lower EOR (HR = 0.110, 95%CI 0.014–0.885, *p* = 0.038), and no Tx before TP (HR = 0.421, 95%CI 0.236–0.751, *p* = 0.0034) were found as independent risk factors of GBM progression (Table 4). The median PFS of GBM patients was 5.7 months with additional resection after ioMRI and 7.6 months without, and was not affected by additional resection after ioMRI (HR = 0.898, 95%CI 0.534–1.483, *p* = 0.67) (Table 5, Figure 2).

**Figure 3:**
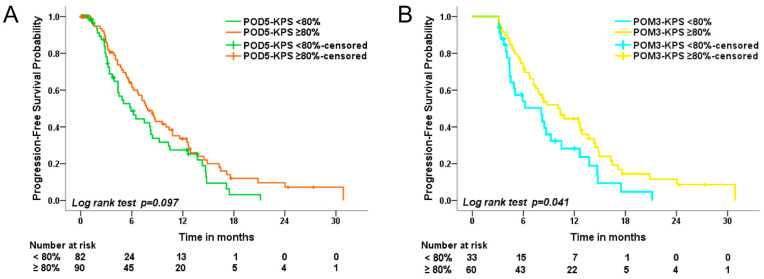
Kaplan–Meier progression-free survival curves for Karnofsky performance status on the fifth postoperative day and in the third postoperative month. This figure illustrates the Kaplan–Meier survival analysis for Karnofsky performance status (**A**) on the fifth postoperative day (POD5-KPS) and (**B**) in the third postoperative month (POM3-KPS). We obtained the follow-up data for POD5-KPS and POM3-KPS from 172 cases and 93 cases, respectively. We compared the progression-free survival of patients with a KPS ≥ 80% to that of patients with a KPS < 80%.

Regarding all 172 cases, the Kaplan–Meier survival analysis on POD5 showed no significant difference between patients with KPS < 80% and those with KPS ≥ 80% (median PFS 5.9 months vs. 7.9 months, *p* = 0.097) (Figure 3A). However, among patients with a PFS over three months (93 patients), Kaplan–Meier survival analysis in POM3 showed a significantly longer PFS for patients with a KPS ≥ 80% (median PFS 8.1 months vs. 10.3 months, *p* = 0.041) (Figure 3B).

### 3.5. Karnofsky Performance Status

Higher age, female gender, longer duration of surgery, higher blood loss, larger craniotomy size, larger tumor volume, lower EOR, and lower preoperative KPS were related to a lower KPS on the POD5 (POD5-KPS). However, preoperative KPS (partial rank correlation coefficient (PRCCI) = 0.460, *p* < 0.0001) and EOR (PRCC = 0.210, *p* = 0.0067), were independently associated with POD5-KPS when controlled for other variates (Table 6).

Additionally, greater age, longer duration of surgery duration, higher blood loss, larger tumor volume, additional resection after ioMRI, lower preoperative KPS, and lower POD5-KPS were associated with a lower POM3-KPS. POD5-KPS (PRCC = 0.589, *p* < 0.0001) was independently associated with POM3-KPS when controlled for other related variates. Furthermore, greater age was associated with worse KPS in POM3, with borderline significance, when controlled for other related variates (PRCC = −0.173, *p* = 0.070).

### 3.6. Surgery-Related Functional Deficits

**Figure 4:**
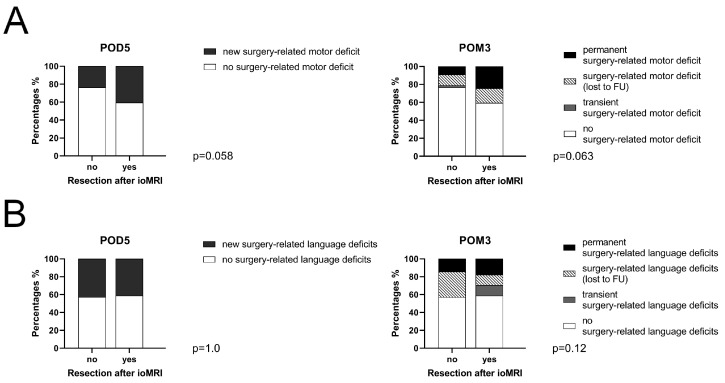
Surgery-related motor and language deficits. This figure shows transient (POD5: on the fifth postoperative day) and permanent (POM3: in the third postoperative month) surgery-related motor deficits (**A**) and surgery-related language deficits in cases with or without additional resection after intraoperative magnetic resonance imaging (ioMRI), including cases lost to follow-up (FU) in POM3 (**B**).

This study included 156 cases of motor-eloquent GBM. A total of 37 cases underwent additional resection after ioMRI, of which 15 (40.5%) showed new surgery-related deficits at POD5, persisting in nine cases (24.3%) in POM3. Among 119 cases without additional resection after ioMRI, new motor deficits at POD5 were reported in 28 (23.5%) cases, including 11 (9.2%) cases of persisting motor deficits in POM3. No significant difference between patients with motor-eloquent GBM was found, when comparing rates of surgery-related motor deficits in cases with and without additional resection after ioMRI at POD5 (*p* = 0.058) or in POM3 (*p* = 0.063) (Table 7, Figure 4A).

A total of 59 cases underwent resection of language-eloquent GBM. Of these, 17 underwent additional resection after ioMRI, including 7 (41.2%) cases showing decreased language function at POD5. In three cases, language deficits persisted in POM3 (17.6%). In 42 cases, no additional resection was performed after ioMRI. In 18 cases (42.9%), language function deteriorated after surgery, which was persistent in six cases (14.3%) in POM3. We found no significant differences in surgery-related aphasia among cases with and without additional resection after ioMRI at POD5 (*p* = 1.0) or in POM3 (*p* = 0.12) (Table 8, Figure 4B).

## 4. Discussion

The present study shows that GBM resection aided by ioMRI could clearly increase the GTR rate from 78.5% to 93.0% (*p* = 0.0002). Mean EOR increased from 93.9% to 98.3%, with residual tumor volume decreasing from 1.3 ± 4.2 cm^3^ to 0.6 ± 2.5 cm^3^. Consequently, we observed no significant difference in residual tumor volume on postoperative MRI between patients with and without additional resection after ioMRI (0.3 ± 1.6 cm^3^ vs. 0.7 ± 2.8 cm^3^, *p* = 0.41). For GBM, GTR is significantly associated with PFS [10]. However, the increased EOR and increased GTR rates accomplished by resection after ioMRI did not transfer to PFS in patients with additional resection since ioMRI can only improve the EOR up to the same EOR as the group without additional resection. It is noteworthy that without the aid of ioMRI, complete resection was achieved in most cases (68.0%) in this study. Senft et al. demonstrated that the application of ioMRI correlated with a significantly higher rate of complete tumor resection in a prospective study with 58 patients [18]. This study found no significant benefits of ioMRI regarding PFS (*p* = 0.083) [18]. A recent meta-analysis on ioMRI by Lo et al. also showed an increase in EOR by 7% in cases of high-grade glioma but failed to improve PFS [3]. Shah et al. found that an increase in EOR and GTR rates of GBM after ioMRI did not correlate with overall survival [4].

Further analysis in our study identified preoperative KPS (*p* = 0.031) and lower EOR (*p* = 0.038) as independent factors indicating lower PFS, which matches with previous studies [15,27,28]. Furthermore, postoperative Tx was confirmed as a significant factor regarding PFS (*p* = 0.0034) [29,30].

Moreover, ioMRI’s cost effectiveness should be considered, as prolonged surgical time and MRI-compatible surgical equipment are required [31,32,33]. Therefore, we aimed to identify subgroups that may especially benefit from ioMRI. In our analysis, recurrent GBM and tumor volume less than 15 cm^3^ were independent negative factors for complete tumor resection before ioMRI. Oringer et al. compared preoperative tumor volume, postoperative tumor volume, and EOR and showed a lower EOR in tumor volumes <10.0 cm while showing increasing residual tumor volume with increasing preoperative tumor volume [34]. Therefore, smaller tumor volume can be considered a risk factor for lower EOR. However, residual tumor volumes are lower in this subgroup, which is an independent favorable factor regarding tumor recurrence and overall survival [35,36].

KPS is commonly used to assess the clinical status of patients suffering from brain tumors, indicating that a worse status is related to a poorer survival rate [15,16]. Previous reports have shown a significant correlation between preoperative KPS and PFS [15,27]. KPS, on average, was stable or declined after surgery in most studies [8,17,29]. Ushio et al. reported an improvement in mean KPS from 78% preoperatively to 83% at discharge in cases in which GTR was achieved [37]. However, EOR’s effect on KPS, especially regarding the use of ioMRI, has rarely been analyzed. Our results confirmed that a higher preoperative KPS was associated with a longer PFS (Table 5). In addition, KPS ≥ 80% in POM3 was correlated with a longer PFS (Figure 3). A higher EOR was independently associated with a better KPS at POD5 but was not associated with KPS in POM3 (Table 6).

The present study shows that ioMRI enables neurosurgeons to confirm EOR and helps to improve EOR in case of residual tumor. However, the necessity of preservation of function is primary and more essential than GTR. ioMRI supplies imaging to update neuronavigation, correct for brain shift and tumor location, and provide additional information, such as early ischemia, hemorrhage, and tumor progression. However, brain shift is generally limited to a few millimeters, which means the maximum volume of additional resection after ioMRI is limited to a few cubic centimeters [38,39]. Regarding surgery-related functional deficits, we found a trend towards higher rates of motor deficits in cases with additional resection after ioMRI (*p* = 0.058) (Table 7, Figure 4A). Such data should always be taken into consideration when it comes to intraoperative decision making regarding further resection after ioMRI. In a setting with the highest technological standards, the role of ioMRI should be reconsidered, and its potential should be focused on functional protection and improving quality of life rather than purely pursuing a complete resection.

One potential limitation of the present study, based on single-institution data, is selection bias. However, this study reports a consecutive series of patients, and a censoring rate lower than 50% and events per variate over 13 matched the requirements of multivariate Cox proportional regression analyses [40]. Detailed histopathological findings, including IDH1 mutation status and MGMT methylation status were not considered and controlled regarding clinical outcome. This must be considered a potential limitation and bias when interpreting the findings of this study. In addition, the surgeons’ knowledge of the availability of ioMRI must be taken into account as potential bias, as ioMRI was routinely performed on all patients included in this study. A multi-institutional analysis with an even larger study cohort could allow for a more general conclusion on the value and the best approach to implement ioMRI in clinical routine.

## 5. Conclusions

Implementing ioMRI during GBM resection improves EOR and GTR and considerably lowers residual tumor burden. Consequently, ioMRI helped to achieve comparable PFS in patients with unexpected tumor residual compared to patients with complete tumor removal before the intraoperative scan. ioMRI did not impact postoperative KPS. We identified lower EOR, lower preoperative KPS and no Tx before TP as individual risk factors for worse PFS in general. Our results further suggest that ioMRI is most beneficial in cases of recurrent GBM and tumor volume less than 15 cm^3^, as we identified these cases with overall low rates of GTR.

## Figures and Tables

**Table 1:** This table illustrates the general information of 172 glioblastoma cases, including patient characteristics and surgery-related data. We estimated ioMRI-related surgery pause time based on the duration of CO_2_-measurement-pause in each case’s anesthesia report. We recorded the duration of ioMRI scans from the beginning of the first sequence to the end of the last sequence. We calculated the ratios of scan time to pause time, scan time to surgery time, and pause time to surgery time per case. Median progression-free survival (PFS) was illustrated with a 95% confidence interval (CI). We obtained preoperative Karnofsky performance status (Preop-KPS) and KPS on the fifth postoperative day (POD5-KPS) and in the third postoperative month (POM3-KPS) as well as the change from Preop-KPS to POD5-KPS and the change between Preop-KPS to POM3-KPS.

Patient Characteristics & Surgical Factors		ioMRI		Clinical Outcomes	
**Age** **(mean ± SD)**	60.1 ± 12.8	ioMRI-related surgery pause time (mean ± SD min)	45.2 ± 8.5	Median PFS (months)	7.5 (95%CI 5.84–9.10)
**Gender** **(male/female)**	119/53	Duration of ioMRI scan (mean ± SD min)	26.9 ± 7.9	Preop-KPS (median, range)	90(40–100)
**Newly diagnosed/Recurrence**	119/53	ioMRI scan time/Surgery pause time (mean ± SD%)	59.4 ± 13.8	POD5-KPS (median, range)	80(10–100)
**Duration of surgery** **(mean ± SD min)**	254.7 ± 65.6	ioMRI scan time/Duration of surgery (mean ± SD%)	11.2 ± 4.0	POM3-KPS (median, range)	80(30–100) (*n* = 117)
**Blood loss** **(mean ± SD mL)**	654.1 ± 601.2	Surgery pause time/Duration of surgery (mean ± SD%)	18.8 ± 5.6	KPS change Preop to POD5 (median, range)	0(−60–20)
**Size of craniotomy** **(mean ± SD cm^2^)**	36.8 ± 14.5	Number of sequences (median, range)	9(2–14)	KPS change Preop to POM3 (median, range)	0(−50–30) (*n* = 117)

**Table 2:** Comparison of postoperative adjuvant radiochemotherapy in patients with or without additional resection after ioMRI. This table illustrates the data on postoperative adjuvant therapy (Tx) from 172 glioblastoma cases before tumor progression (TP). The number of cases undergoing radiotherapy (RTx) and chemotherapy (CTx), as well as cases lost to follow-up (FU) and undergoing best-supportive care are demonstrated in the categories with or without additional resection after ioMRI.

	Tx before TP (*n* = 103)			No Tx before TP (*n* = 46)	No Tx before Lost to FU (*n* = 23)	*p*-Value ^+^
	Only RTx	Only CTx	RTx & CTx			
**With additional resection (*n* = 43)**	1 (2.3%)	5 (11.6%)	15 (34.9%)	15 (34.9%)	7 (16.3%)	0.51
**Without additional resection (*n* = 129)**	9 (7.0%)	19 (14.7%)	54 (41.9%)	31 (24.0%)	16 (12.4%)	

^+^ Fisher’s exact test.

**Table 3:** Volumetric analysis with and without secondary resection after ioMRI. This table shows the volumetric analysis with and without additional resection after ioMRI, including preoperative tumor volume, tumor volume of first resection, extent of resection before ioMRI (EOR I), residual volume measured at ioMRI, tumor volume of additional resection assisted by ioMRI, the postoperative residual volume, and EOR between preoperative MRI and postoperative MRI (EOR III). We recorded differences in EOR between EOR I and EOR III as EOR II. We distinguished gross total resection (GTR) from subtotal resection (STR) by an EOR ≥ 95%.

	With Additional Resection	Without Additional Resection	*p*-Value	Total
**Number (%)**	43(25.0%)	129(75.0%)	-	172
**Preoperative tumor volume** **(mean ± SD cm^3^)**	28.8 ± 32.9	34.1 ± 34.2	0.37 ^∆^	32.8 ± 33.9
**First resection volume** **(mean ± SD cm^3^)**	25.5 ± 29.9	33.4 ± 34.0	0.18 ^∆^	31.4 ± 33.1
**EOR I** **(mean ± SD%)**	81.8 ± 19.6	98.0 ± 9.0	<0.0001 ^∆^	93.9 ± 14.3
**GTR rate before ioMRI** **(GTR/STR)**	37.2% (16/27)	92.2% (119/10)	<0.0001 ^+^	78.5% (135/37)
**Residual volume on ioMRI** **(mean ± SD cm^3^)**	3.3 ± 6.5	0.7 ± 2.8	0.0003 ^∆^	1.3 ± 4.2
**Volume of additional resection** **(mean ± SD cm^3^)**	3.0 ± 6.1	-	-	0.7 ± 3.3
**EOR II** **(mean ± SD%)**	17.3 ± 18.8	-	-	4.3 ± 12.0
**Residual volume postoperatively** **(mean ± SD cm^3^)**	0.3 ± 1.6	0.7 ± 2.8	0.41 ^∆^	0.6 ± 2.5
**EOR III** **(mean ± SD%)**	99.1 ± 4.9	98.0 ± 9.0	0.45 ^∆^	98.3 ± 8.2
**GTR rate postoperatively** **(GTR/STR)**	95.3% (41/2)	92.2% (119/10)	0.73 ^+^	93.0% (160/12)

^∆^ *T*-test. ^+^ Fisher’s exact test.

**Table 4:** Analyses of variables affecting gross total resection of glioblastoma. This table illustrates the univariate analysis and logistic regression analysis of variables contributing to subtotal resection (STR) when gross total resection (GTR) was considered achievable. The number of cases is 157. We compare the median and interquartile range (IQR) of age and craniotomy size. For univariate analysis, we counted and analyzed gender distribution, recurrent glioblastoma (GBM), tumor volume (grouped by 15 cm^3^), and tumor location in eloquent areas. We present logistic regression analysis results as odds ratio (OR) and 95% confidence interval (CI).

	Univariate Analysis			Logistic Regression		
	GTR (*n* = 133)	STR (*n* = 24)	*p*-Value	OR	95%CI	*p*-Value
**Age, median ± IQR**	60.0 ± 16.0	59.5 ± 13.0	0.68 ^∆^	-	-	-
**Size of craniotomy, median ± IQR (cm^2^)**	37.0 ± 20.0	32.5 ± 19.0	0.26 ^∆^	-	-	-
**Gender, *n* (%)**						
**Male (*n* = 108)**	89(82.4%)	19(17.6%)	0.34 ^+^	-	-	-
**Female (*n* = 49)**	44(89.8%)	5(10.2%)				
**Recurrent GBM, *n* (%)**						
**Yes (*n* = 46)**	32(69.6%)	14(30.4%)	0.0013 ^+^	3.047	1.165–7.974	0.023
**No (*n* = 111)**	101(91.0%)	10(9.0%)		1.000		
**Tumor volume, *n* (%)**						
**<** **15 cm^3^ (*n* = 72)**	54(75.0%)	18(25.0%)	0.0032 ^+^	3.031	1.062–8.651	0.038
**≥15 cm^3^ (*n* = 85)**	79(92.9%)	6(7.1%)		1.000		
**Eloquent area, *n* (%)**						
**Yes (*n* = 144)**	123(85.4%)	21(14.6%)	0.42 ^+^	-	-	-
**No (*n* = 13)**	10(76.9%)	3(23.1%)				

^∆^ *T*-test. ^+^ Fisher’s exact test.

**Table 5:** Univariate and multivariate Cox proportional regression analyses of the progression-free survival of glioblastoma. This table shows the uni- and multivariate Cox proportional regression analyses of progression-free survival. The number of cases is 172, and the number of events is 108. The hazard ratio (HR) and the 95% confidence interval (CI) are shown. Age, gender, recurrent glioblastoma (GBM), preoperative tumor volume, postoperative extent of resection, preoperative Karnofsky performance status (KPS), adjuvant therapy before tumor progression (TP), and additional resection after intraoperative MRI (ioMRI) are included.

	Univariate Analysis			Multivariate Analysis		
	HR	95%CI	*p*-Value	HR	95%CI	*p*-Value
**Age**	1.017	1.001–1.033	0.037	1.015	0.998–1.033	0.090
**Gender (male/female)**	1.005	0.661–1.527	0.98	1.264	0.804–1.986	0.31
**Recurrent GBM (yes/no)**	0.995	0.664–1.491	0.98	0.800	0.488–1.311	0.38
**Tumor volume (cm^3^)**	1.001	0.996–1.006	0.81	0.999	0.993–1.005	0.84
**Extent of resection (%)**	0.187	0.025–1.397	0.10	0.110	0.014–0.885	0.038
**Resection after ioMRI (yes/no)**	1.165	0.738–1.840	0.51	0.898	0.543–1.483	0.67
**Preoperative KPS**	0.981	0.966–0.995	0.0081	0.983	0.967–0.998	0.031
**Adjuvant therapy before TP (yes/no)**	0.475	0.295–0.766	0.0023	0.421	0.236–0.751	0.0034

**Table 6:** Correlation analysis of Karnofsky performance score (KPS) on the fifth postoperative day and in the third postoperative month after surgery for glioblastoma. This table illustrates the results of the Kendall-rank correlation analysis on the Karnofsky performance status on the fifth postoperative day (POD5-KPS) and in the third postoperative month (POM3-KPS). Age, gender, recurrent tumor, surgery duration, blood loss, craniotomy size, tumor volume, additional resection after ioMRI, extent of resection (EOR) postoperatively, ioMRI-related surgery pause time, duration of ioMRI scan, and preoperative KPS (Preop-KPS) were analyzed.

	POD5-KPS (*n* = 172)					POM3-KPS (*n* = 117)				
		Rank Correlation Coefficient	*p*-Value	Partial Rank Correlation Coefficient	*p*-Value		Rank Correlation Coefficient	*p*-Value	Partial Rank Correlation Coefficient	*p*-Value
**Age (mean ± SD)**	60.1 ± 12.8	−0.162	0.0036	−0.100	0.20	59.0 ± 11.2	−0.220	0.0013	−0.173	0.070
**Gender (male/female)**	119/53	−0.155	0.021	−0.110	0.16	84/33	−0.079	0.33	-	-
**Recurrent tumor (yes/no)**	53/119	−0.042	0.53	-	-	41/76	0.039	0.64	-	-
**Duration of surgery** **(mean ± SD min)**	254.7 ± 65.6	−0.214	0.0001	−0.054	0.49	246.7 ± 61.7	−0.173	0.011	−0.042	0.66
**ioMRI-related surgery pause time** **(mean ± SD min)**	45.2 ± 8.5	0.082	0.14	-	-	44.7 ± 9.3	0.073	0.29	-	-
**Duration of ioMRI scan** **(mean ± SD min)**	26.9 ± 7.9	0.005	0.94	-	-	26.3 ± 8.8	0.079	0.25	-	-
**Blood loss (mean ± SD mL)**	654.1 ± 601.2	−0.209	0.0002	−0.062	0.43	582.1 ± 480.1	−0.137	0.050	0.009	0.93
**Size of craniotomy** **(mean ± SD cm^2^)**	36.8 ± 14.5	−0.220	<0.0001	−0.119	0.13	36.3 ± 14.3	−0.121	0.075	-	-
**Tumor volume (mean ± SD cm^3^)**	32.8 ± 33.9	−0.244	<0.0001	−0.023	0.77	28.2 ± 31.9	−0.201	0.0028	−0.055	0.57
**Resection after ioMRI (no/yes)**	129/43	−0.075	0.27	-	-	87/30	−0.225	0.0061	−0.137	0.15
**EOR after ioMRI (mean ± SD%)**	98.3 ± 8.2	0.211	0.0013	0.210	0.0067	98.5 ± 8.0	0.113	0.16	-	-
**Tumor progression (yes/no)**	0/172	-	-	-	-	24/93	0.155	0.059	-	-
**Preop-KPS (median, range)**	90(40–100)	0.511	<0.0001	0.460	<0.0001	90 (40–100)	0.471	<0.0001	0.053	0.58
**POD5-KPS (median, range)**	80(10–100)	1	-	-	-	80 (30–100)	0.708	<0.0001	0.589	<0.0001
**POM3-KPS (median, range)**	-	-	-	-	-	80 (30–100)	1	-	-	-

**Table 7:** Comparison of surgery-related motor deficits in patients with motor-eloquent glioblastoma with and without additional resection after ioMRI. This table illustrates the postoperative motor status on the fifth postoperative day (POD5) and in the third postoperative month (POM3) regarding surgery-related motor deficits (SRMD), including cases lost to follow-up (FU) in POM3.

	With Additional Resection (*n* = 37)				Without Additional Resection (*n* = 119)				*p*-Value ^+^
**POD5**	**New motor deficits**			**No SRMD**	**New motor deficits**			**No SRMD**	0.058
	15 (40.5%)				28 (23.5%)				
**POM3**	**Permanent**	**Transient**	**Lost to FU**	22 (59.5%)	**Permanent**	**Transient**	**Lost to FU**	91 (76.5%)	0.063
	9 (24.3%)	0 (0%)	6 (16.2%)		11 (9.2%)	3 (2.5%)	14 (11.8%)		

^+^ Fisher’s exact test.

**Table 8:** Comparison of surgery-related language deficits in patients with language eloquent glioblastoma with and without additional resection after ioMRI. This table illustrates the postoperative status of language function on the fifth postoperative day (POD5) and in the third postoperative month (POM3) regarding surgery-related language deficits (SRLD), including cases lost to follow-up (FU) in POM3.

	With Additional Resection (*n* = 17)				Without Additional Resection (*n* = 42)				*p*-Value ^+^
**POD5**	**New language deficits**			**No SRLD**	**New language deficits**			**No SRLD**	1.0
	7 (41.2%)				18 (42.9%)				
**POM3**	**Permanent**	**Transient**	**Lost to FU**	10 (58.8%)	**Permanent**	**Transient**	**Lost to FU**	24 (57.1%)	0.12
	3 (17.6%)	2 (11.8%)	2 (11.8%)		6 (14.3%)	0 (0%)	12 (28.6%)		

^+^ Fisher’s exact test.

## Data Availability

In the interest of patient privacy, all the collected raw data of individual cases are imparticipable. The anonymous datasets used and analyzed during the current study are available from the corresponding author on reasonable request.
